# Taking a closer look at visual search: Just how feature-agnostic is singleton detection mode?

**DOI:** 10.3758/s13414-018-01642-y

**Published:** 2019-01-02

**Authors:** Anthony M. Harris, Oscar Jacoby, Roger W. Remington, Susan L. Travis, Jason B. Mattingley

**Affiliations:** 1 Queensland Brain Institute, University of Queensland, St Lucia, Australia; 2 Institute of Cognitive Neuroscience, University College London, London, UK; 3 School of Psychology, University of Queensland, St Lucia, Australia; 4 Department of Psychology, University of Minnesota, Minneapolis, MN USA

**Keywords:** Visual search, Selective attention, Electrophysiology

## Abstract

Singleton detection mode is a state in which spatial attention is set to prioritize any objects that differ from all other objects present on any feature dimension. Relatively little research has been devoted to confirming the consequences such a search mode has for stimulus processing. It is often implied that when observers employ singleton detection mode, all singletons capture attention equally, and when observers search for a single feature, only that feature captures attention. The experiment presented here contradicts these implications. We had observers search for colored singleton targets preceded by spatially uninformative colored singleton cues, and we recorded stimulus-evoked neural responses using electroencephalography (EEG). When observers had to respond to targets defined by two possible colors (a task intended to encourage singleton detection mode), cue validity effects were apparent for both target-color cues and irrelevant-color cues, and these effects were accompanied by an N2pc in the EEG data. Importantly, however, the target-color cues evoked significantly larger cue validity effects and N2pc components than did the irrelevant-color cues. In contrast, when observers had to respond to targets defined by one color (a task intended to encourage feature search mode), only cues of that color evoked a cue validity effect. Interestingly, the N2pcs produced by irrelevant cues did not differ between feature and singleton search, suggesting that the behavioral difference was not due to different attentional orienting. Rather, we suggest that behavioral singleton capture is due to a diminished same-location cost being produced by irrelevant-color cues.

How do we find things we are looking for? This deceptively simple question has been the subject of considerable research (for selected reviews, see Awh, Belopolsky, & Theeuwes, 2012; Corbetta, Patel, & Shulman, 2008; Desimone & Duncan, 1995; Evans et al., 2011; Gilbert & Li, 2013; Wolfe, 1994; Wolfe & Horowitz, 2017). Much of this research has attempted to identify the search modes available to us during visual search tasks. For example, we know that observers can selectively prioritize the processing of information at specific locations of interest in the visual field, a mechanism referred to as *spatial attention* (e.g., Evans et al., 2011). Observers can also selectively prioritize the processing of any objects with a specific feature value, such as the color red, a mechanism referred to as *feature-based attention*, or in the case of visual search, *feature search* (Maunsell & Treue, 2006; Pashler 1988). There is also some evidence that people can prioritize any unique objects (“singletons”) that differ on some feature dimension from a set of homogeneous objects, a search strategy commonly referred to as *singleton detection mode* (e.g., Bacon & Egeth, 1994). The key motivation for the present study was to gain a better understanding of singleton detection mode and the degree to which it is distinguishable from feature search mode.

A number of studies have provided support for the existence of singleton detection mode (e.g., Bacon & Egeth, 1994; Eimer & Kiss, 2010; Folk & Anderson, 2010; Harris, Becker, & Remington, 2015; Lamy, Carmel, Egeth, & Leber, 2006; Lamy & Egeth, 2003; Leber & Egeth, 2006). Many of these studies have employed a spatial-cueing paradigm (Folk, Remington, & Johnston, 1992) in which a search array is presented shortly after a peripheral spatial cue, with attentional capture being assessed by the impact of the cue on responses to the target. For example, Folk and Anderson (2010) presented a colored target character (an “X” or an “=”) among three white nontarget characters, preceded by a spatial cue consisting of a set of colored dots among three sets of white dots. The location of the spatial cue was independent of the location of the target character, meaning that the cue gave no information about where the subsequently presented target might appear. The target character could be either red or green, and the cue could be red, green, *or* blue. Attentional capture by the cues was assessed by examining the *cue validity effect*, or the extent to which behavioral responses to the target character were faster for validly cued targets (targets that appeared at the same location as the cue) than for invalidly cued targets (targets that appeared at a different location from the cue). In Folk and Anderson’s Experiment 1, in which participants searched for red and green targets, all three cue colors produced a cue validity effect. Since observers knew that the target character they were looking for would never be blue, the fact that the blue cues still produced a cue validity effect suggests that observers were indeed adopting a singleton detection mode (i.e., setting themselves to prioritize any color singletons) rather than adopting a two-feature search mode (i.e., setting themselves to prioritize only red or green objects).^1^ In a second experiment, participants reported the identity of characters defined by one color (either red or green, which varied between participants), and the instructions were intended to encourage participants to employ a feature search mode (e.g., Folk & Remington, 1998). Under these conditions, cues of the same color as the participant’s target color produced a cue validity effect, but cues of the other two colors did not. This finding indicates that the observed influence of the irrelevant blue cues in Experiment 1 was not a purely stimulus-driven effect, since it could be overcome by different task instructions.

Eimer and Kiss (2010) used electroencephalography (EEG) to provide a more detailed picture of the stimulus processing stages influenced by singleton detection mode. They used a spatial-cueing paradigm similar to that used by Folk and Anderson (2010), but in addition to measuring cue validity effects, they also measured an event-related brain potential (ERP) component called the N2pc, widely interpreted as an electrophysiological marker of attentional capture (e.g., Luck, 2012; Woodman & Luck, 2003). In line with the findings of Folk and Anderson, when observers searched for any color singleton, target-color and irrelevant-color cues produced a cue validity effect on reaction times (RTs) to the target items, despite the irrelevant color never being associated with a target. Interestingly, however, the cue validity effect associated with the irrelevant-color cues was significantly smaller than that associated with the target-color cues. This finding suggests that, even though all cues may have captured attention to some extent, priority was given to target-color cues at some stage of processing. A slightly different pattern of results was apparent when cue processing was indexed via the N2pc. Here, both target-color and irrelevant-color cues evoked a reliable N2pc, again consistent with the notion that all cues captured attention to their location. There was, however, no reliable difference in the N2pc amplitudes or latencies between the two cue conditions, although there was a nonsignificant trend for target-color cues to evoke a slightly larger and earlier N2pc than irrelevant-color cues. Given the reasonably small sample size in the study by Eimer and Kiss (12 participants were included in their final analyses), it is difficult to know whether the discrepancy was due to noise in the behavioral or ERP results or instead reflects a true dissociation between their behavioral and electrophysiological indices of attentional capture.

Recently, Carmel and Lamy (2015) argued that the patterns of results observed in attentional capture experiments may in fact reflect the summation of three different cognitive factors: the first, feature-based attentional capture by stimuli that possess target-defining properties; the second, attentional capture by singleton stimuli under conditions in which the target is a unique singleton (even if it also possesses a consistent feature, such as a red target that is always presented among white nontargets); and the third, a same-location cost, such that stimuli that do not match the target features slow the processing of a target that is subsequently presented at their location. Through a series of experiments, Carmel and Lamy (2015) showed that when the target has a defining property but is also a singleton, the typically observed absence of a cue validity effect for irrelevant-color singleton cues (e.g., Folk & Anderson, 2010, Exp. 2, described above) is due to the summation of a positive singleton capture effect with a negative same-location cost. This is consistent with the findings of Eimer and Kiss (2010), described above, who observed no cue validity effect for irrelevant-color cues in search for a single target color, although a significant N2pc was elicited by these cues, consistent with the occurrence of singleton capture that was masked in behavior by a same-location cost.

The findings of Carmel and Lamy (2015) raise interesting questions about singleton detection mode as it has typically been observed in behavior. Under singleton detection mode, all cues produce cue validity effects even if they do not possess a target feature (but see Harris et al., 2015). Under the Carmel and Lamy (2015) framework, it is unclear why this would be the case. As we noted above, Carmel and Lamy (2015) proposed that singleton capture occurs whenever the target is a singleton, even if targets are defined only by a single feature (e.g., red). If this is the case, it is not clear why having two target features rather than one should change the observed cue validity effect for cues that do not possess a target feature. To give a concrete example, it is unclear why an irrelevant blue singleton cue should produce no cue validity effect in search for a red singleton target, but produce a robust cue validity effect in search for red and green singleton targets, when in both of these cases the targets are singletons, the blue cue possesses no target features, and the conditions required to produce a same-location cost should be matched. One possibility is that with more than one target feature the contribution of singleton capture to the cue validity effect is enhanced, such that it overwhelms the same-location cost in order to produce a cue validity effect. Alternatively, search for more than one target may somehow diminish the magnitude of the same-location cost without affecting singleton capture.

Here we sought to resolve some of these questions about singleton detection mode. We had two main aims. First, we sought to determine whether the specific features associated with each of the possible target stimuli are enhanced in singleton detection mode, consistent with the behavioral results of Eimer and Kiss (2010), or whether all cues are treated equally, consistent with their N2pc results. The lack of an enhanced N2pc component for target-colored cues in Eimer and Kiss’s study *might* suggest that the behavioral results reflect enhancement of the target color at a postperceptual stage of processing, not the initial capture of attention. Alternatively, it might simply represent a Type 2 error, given the relatively small sample size used in that study (*N* = 12 included datasets). To give us more statistical power, we increased our sample size to 36 participants, yielding a better than 80% chance of detecting a moderate effect size (Cohen’s *d*_z_ = 0.5). If target features receive additional enhancement under singleton detection mode, in addition to the enhancement received by all singletons, then we would expect to observe both a larger cue validity effect and a larger N2pc to target-colored than to non-target-colored cues under singleton detection mode. However, if the larger cue validity effect produced by target-colored cues under singleton detection mode is not attentional in nature, then we should replicate the results of Eimer and Kiss and show no difference between the N2pc magnitudes for target-colored and non-target-colored cues.

The second aim of our study was to examine whether the emergence of a cue validity effect for irrelevant-color cues in singleton search is due to an enhancement of singleton capture itself. This may be due to an emergence of singleton capture that is not present in feature search, or because the singleton capture that is present in “feature search” (when the target is also a singleton) is enhanced such that it overwhelms any potential contribution of a same-location cost. Alternatively, cue validity effects for irrelevant-color cues may emerge with no change in the degree of singleton capture, because the same-location cost is removed or diminished under these conditions. If singleton capture is enhanced under singleton detection mode, the N2pc component elicited by irrelevant-color cues should be larger when searching for two targets (singleton search) than when searching for one target (feature search). However, if behavioral singleton capture effects are due to a diminished contribution of the same-location cost, we would expect the N2pcs to irrelevant-color cues to be the same under the two search conditions. In addition to using a behavioral cue validity effect and the N2pc component to measure cue processing, we also analyzed the Pd component, an ERP measure of attentional suppression or disengagement (Hickey, Di Lollo, & McDonald, 2009; Sawaki, Geng, & Luck, 2012; Sawaki & Luck, 2013; but see Livingstone, Christie, Wright, & McDonald, 2017). This allowed us to examine differences between the search modes at this stage of processing, which could potentially explain any differences in behavior that were not associated with a difference in the N2pc.

## Materials and method

### Participants

In total, 43 staff and students (26 female, 17 male, between 18 and 36 years of age) from the University of Queensland, Australia, took part in the experiment. Seven of these participants were excluded for excessive eye movement violations during the task, as described below. We replaced any excluded participants until we had 36 participants (24 female, 12 male, between 18 and 36 years of age) in our final included dataset, three times as many as had been included in the study by Eimer and Kiss (2010). All participants reported normal or corrected-to-normal vision and normal color vision. The University of Queensland Human Research Ethics Committee approved all procedures. We obtained written informed consent from each participant prior to each testing session. Participants were financially reimbursed for their time.

### Apparatus

Stimulus presentation and response recording were controlled using the Cogent software (Cogent 2000 toolbox: FIL, ICN, and Wellcome Department of Imaging Neuroscience) in Matlab version 8.2 (www.mathworks.com) running on a desktop computer. The visual stimuli were presented against a black background on an LCD monitor at a screen resolution of 1,920 × 1,080 pixels and a refresh rate of 100 Hz. Participants were seated at a viewing distance of 48 cm from the monitor, maintained using a chin rest.

### Stimuli

The stimuli used in this experiment were very similar to those used by Folk and Anderson (2010). The primary difference was that we removed the upper and lower stimulus locations used in the previous study, such that stimuli could now appear only in one of two locations, to the left and right of fixation. We made this change so that every single cue stimulus could be included in our analyses of contralateral ERP components. (This is because stimuli presented at the vertical midline of the visual field do not generate contralateral ERP components.)

**Fig. 1:**
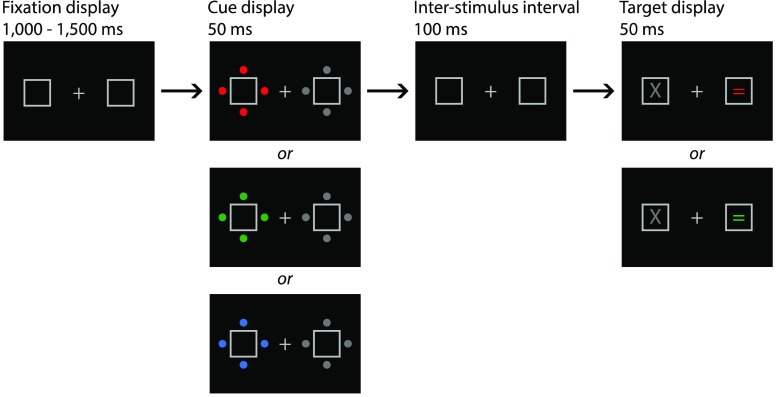
Illustration of the visual stimuli for a participant whose two possible target character colors were red and green. The colored cue could either appear around the same box as the subsequent colored item (valid cues) or around the other box (invalid cues, as in the example above)

Each trial involved a fixation display, a cue display, and a target display (see Fig. 1). The fixation display contained a light gray central fixation plus sign (width and height = 0.4°, line thickness = 0.05°) flanked by light gray placeholder boxes (width and height = 2°, line thickness = 0.05°), centered 5.7° to the left and right of fixation. The cue display included the fixation display as well as four filled circles 0.4° in diameter surrounding each placeholder box (each dot was centered 1.3° from the center of its placeholder box). The circles around one placeholder box were gray, and the circles around the other placeholder box were either red, green, or blue.^2^ The luminance of all four possible cue circle colors was matched at 21.5 cd/m^2^. The target display included the fixation display, as well as an “X” in the center of one of the placeholder boxes and an “=” in the other. One character was gray, and the other character was one of two possible colors. The selection of the two possible target display colors from the three options (red, green, and blue) was counterbalanced across participants. Each character subtended 0.7° in width and was written in Arial font.

### Design and procedure

Participants took part in two separate testing sessions. The stimuli presented during each session were identical; all that differed was the participants’ task. In the singleton search task, participants were instructed to report the identity of any colored character on each trial (i.e., both of the colors presented in the target displays were target colors). We expected these task instructions to cause participants to adopt a singleton search mode. In the feature search task, participants were instructed to report the identity of characters of one of the two possible target display colors only, and to ignore characters in the other color. We refer to these two colors as the *target color* and the *nontarget color*, respectively, in line with the terminology used by Eimer and Kiss (2010). The assignment of the two target display colors to these two conditions was counterbalanced across participants. Note that, during the singleton search task, both of these colors were target colors, so they were collapsed for the analysis of this condition. We refer to the cue color that never appeared in the target display as the *irrelevant color* for both search tasks, again in line with Eimer and Kiss. For the analyses of the feature search condition, the nontarget color and the irrelevant color were collapsed in order to increase statistical power. Analyzing them separately produced equivalent results, consistent with the findings of Eimer and Kiss.

Participants were instructed to keep their eyes fixated on the plus sign in the middle of the display for the duration of each trial and to respond as quickly and accurately as possible. Participants were also informed that sets of gray and colored dots would briefly appear before the target displays, that these dots would not give them any clue as to where the colored item would appear, and that they should try to ignore the dots as best as they could. The order in which the two sessions were completed was counterbalanced across participants. Exploratory analyses that included session order as a between-subjects factor produced no significant effects involving this factor, so the data from the two sessions were collapsed for the reported analyses.

Each trial began with only the placeholder boxes on the screen for 100 ms, followed by the fixation display for a random interval of between 1,000 and 1,500 ms. The cue display then appeared for 50 ms, followed by the fixation display again for 100 ms, then the target display for 50 ms, and finally the fixation display again. Participants made their responses by pressing either the “0” or the “.” key on the numeric keypad of a computer keyboard, for the “X” and “=” characters, respectively (the keys were marked appropriately). If they failed to respond to a target item within 1,500 ms, the phrase “TOO SLOW!” was presented on screen for 300 ms. If participants incorrectly identified the target character, they received the visual feedback “WRONG BUTTON!,” and if they responded to a non-target-color character during the feature search task, they received the visual feedback “WRONG COLOUR!” The next trial began immediately following a response and any required visual feedback.

Each search task consisted of 14 blocks of 96 trials. Each block was further subdivided into 16 “mini-blocks” of six trials. There were two trials for each cue color during each mini-block. Cue location (left, right), target location (left, right), and target identity (“X,” “=”) were counterbalanced within each set of eight mini-blocks. During half of the mini-blocks in each block, all six colored characters were the color that participant had to respond to during the feature search task. In these mini-blocks, participants had to respond on all six trials during both the singleton and feature search tasks. During the other half of the mini-blocks, half of the colored characters were the feature search target color, and the other half were the other possible target display color. Here participants had to respond on all trials during the singleton search task, but on only half of the trials during the feature search task. The two types of mini-blocks were intermixed pseudorandomly within each block, such that there could only be a maximum of two mini-blocks of the same type in a row.

### EEG recording and data analysis

Continuous electroencephalogram (EEG) data were recorded using a BioSemi Active Two system (BioSemi, Amsterdam, Netherlands), digitized at a 1024-Hz sample rate with 24-bit A/D conversion. The 64 active scalp Ag/AgCl electrodes were arranged according to the international standard 10**–**10 system for electrode placement (Oostenveld & Praamstra, 2001) using a nylon head cap. The standard BioSemi reference and ground electrodes were used during recording. Eye movements were monitored using bipolar horizontal electro-oculographic (EOG) electrodes placed at the outer canthus of each eye and bipolar vertical EOG electrodes placed above and below the left eye.

Offline EEG data analysis was performed using purpose-built Matlab scripts. Noisy scalp channels, identified by visual inspection of the data, were replaced by a spherical spline interpolation of the voltages recorded at all other scalp electrodes (an average of 0.3 electrodes per testing session, ranging between 0 and 3). The data for the scalp electrodes were then re-referenced to the average of all 64 scalp electrodes, subjected to a 40-Hz low-pass digital filter, and segmented into epochs from 100 ms before to 400 ms after the onset of each cue display. The average voltage in the 100-ms prestimulus interval was used as a baseline for each epoch. Epochs in which the difference between the maximum and minimum voltage exceeded 50 *μ*V in the HEOG channel, 60 *μ*V in the VEOG channel, or 80 *μ*V in any other channel were automatically rejected in order to remove epochs contaminated by eye movements, blinks, and other artifacts. Seven participants were excluded from further analysis for having more than 30% of epochs rejected for violating these criteria, a threshold we had decided on a priori. An average of 12% of epochs were rejected for violating these criteria in the 36 participants included in the final analyses. We then averaged the accepted epochs together separately for each search task, cue color, and cue location. We then collapsed the data across cue locations by combining all data for electrodes contralateral to the cue (i.e., electrodes left of the midline for cues presented on the right, and electrodes right of the midline for cues presented on the left) and separately combining the data for electrodes ipsilateral to the cue. We then created a difference wave by subtracting the ipsilateral from the contralateral waveforms for electrodes PO7 and PO8. These electrodes are usually chosen for analyses of contralateralized visual-evoked potentials such as the N2pc and Pd components (Luck, 2012).

## Results

### Behavioral results

**Fig. 2:**
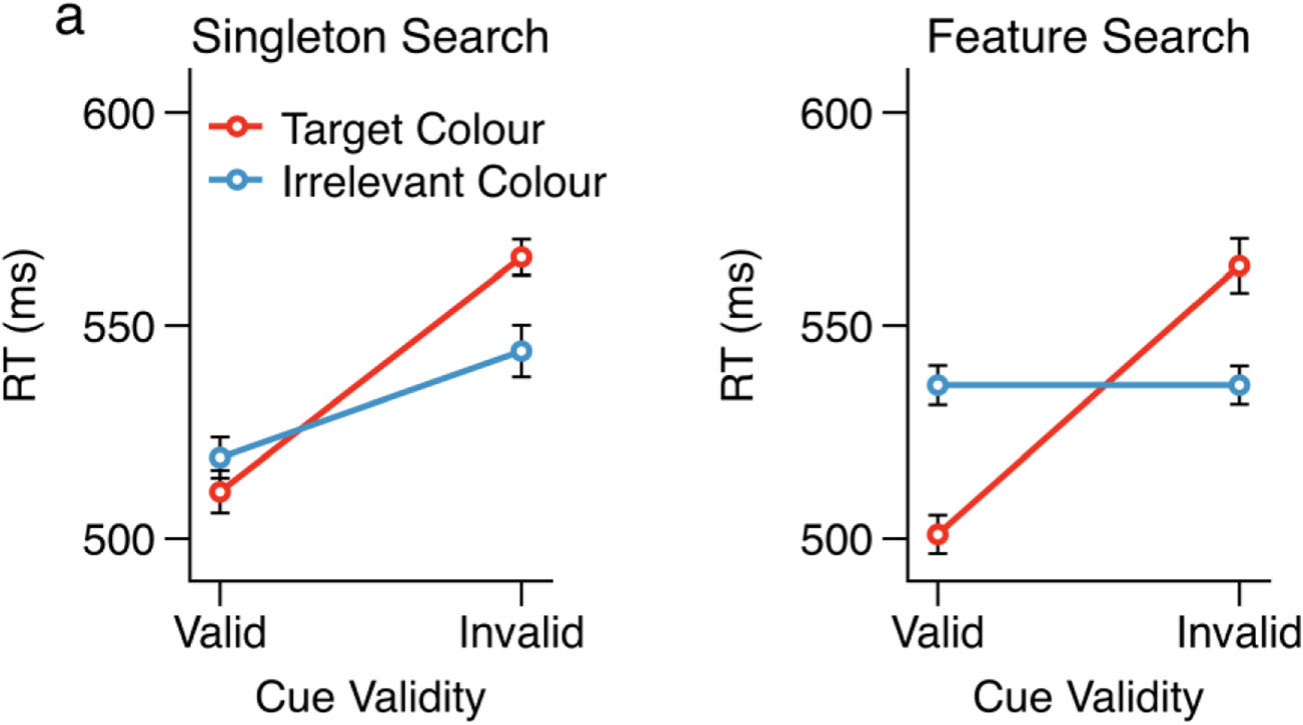
Mean reaction time data as a function of cue validity and cue color, shown separately for each search task. Error bars represent within-subjects confidence intervals (Morey, 2008)

Mean RTs as a function of cue validity and search task are shown in Fig. 2, separately for each search task. For all analyses reported here, we combined the data from the two target colors during the singleton search task and from the two irrelevant colors in the feature search task. We subjected the RT data to a three-way analysis of variance (ANOVA) with the factors search task, cue validity, and cue color. The results revealed a significant three-way interaction, *F*(1, 35) = 23.02, *p* < .001, *η*^2^ = .40. This was followed up with separate two-way ANOVAs for each of the search tasks, with the factors cue validity and cue color.

For the singleton search task (Fig. 2a), we found a significant interaction between cue color and cue validity, *F*(1, 35) = 31.23, *p* < .001, *η*^2^ = .47. Following up this interaction with repeated measures *t* tests comparing the cue validity effects separately for each of the cue conditions revealed significant cue validity effects produced by the target-color cues, *t*(35) = 12.06, *p* < .001, Cohen’s *d*_z_ = 2.01, as well as by the irrelevant-color cue, *t*(35) = 7.04, *p* < .001, Cohen’s *d*_z_ = 1.17. As was observed by Eimer and Kiss (2010), however, the cue validity effect associated with the target-color cues (55 ms) was significantly larger than that associated with the irrelevant-color cue (26 ms), as evidenced by a significant interaction between cue validity and cue color.

For the feature search task (Fig. 2b), we also found a significant interaction between cue validity and cue color, *F*(1, 35) = 106.06, *p* < .001, *η*^2^ = .75. Within-subjects *t* tests revealed a significant cue validity effect associated with the target-color cue (63 ms), *t*(35) = 12.36, *p* < .001, Cohen’s *d*_z_ = 2.06, but no cue validity effect associated with the irrelevant-color cues (0 ms), *t*(35) = 0.16, *p* = .873, Cohen’s *d*_z_ = 0.03. Overall, the mean RTs during the singleton search task (*M* = 536 ms, *SE* = 8 ms) were not significantly different from those during the feature search task (*M* = 535 ms, *SE* = 11 ms), *F*(1, 35) = 0.02, *p* = .903, *η*^2^ = .00, suggesting that the two tasks were roughly equivalent in terms of difficulty.

If the emergence of a cue validity effect for irrelevant-color cues in the singleton search condition was due to a reduction of the same-location cost relative to feature search, we would expect the cue validity effect to be driven by a speeding of valid RTs in singleton search relative to feature search, with no slowing of invalid RTs. Paired-samples *t* tests comparing feature search with singleton search for irrelevant-color cues at valid and invalid locations confirmed this prediction: Irrelevant-color cues presented at valid locations produced significantly faster responses under singleton search than under feature search, *t*(35) = 2.06, *p* = .047, Cohen’s *d*_z_ = 0.34. There was, however, no difference between RTs following irrelevant cues at invalid locations under singleton search and feature search, *t*(35) = 0.86, *p* = .396, Cohen’s *d*_z_ = 0.14.

**Fig. 3:**
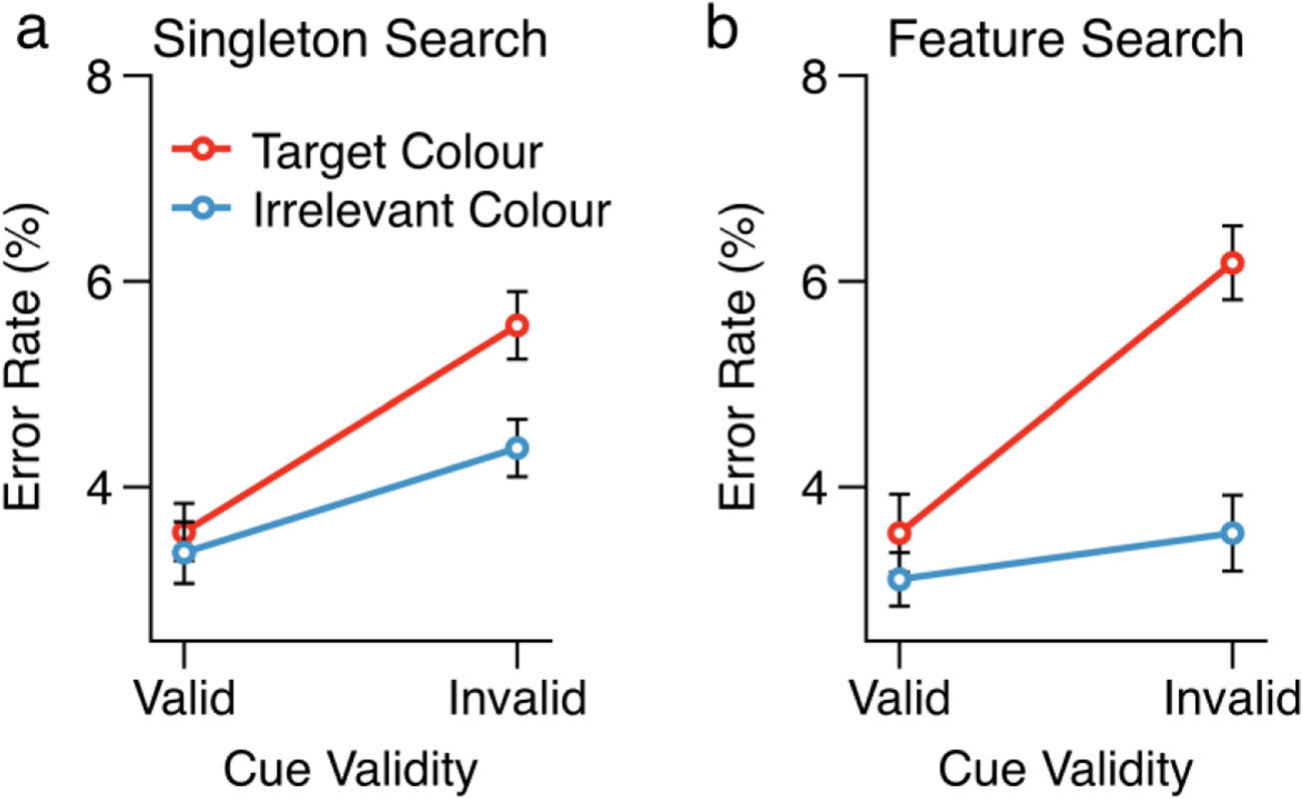
Mean error rate data as a function of cue validity and cue color, shown separately for each search task. Error bars represent within-subjects confidence intervals (Morey, 2008)

We also analyzed the error rates associated with each condition, using the same analyses we had applied to the RT data. The mean error rates are shown in Fig. 3, separately for each search task. There was no significant three-way interaction between search task, cue condition, and cue validity, *F*(1, 35) = 2.13, *p* = .153, *η*^2^ = .06. Planned follow-up comparisons, however, did show a pattern of effects that closely mirrored those observed in the RT data. (It should be noted that error rates are reported for completeness, but the contingent capture task employed here was designed to produce effects on RTs, not error rates.) For the singleton search task (Fig. 3a), we observed significant cue validity effects for both the target-color cues, *t*(35) = 5.70, *p* < .001, Cohen’s *d*_z_ = 0.95, and the irrelevant-color cue, *t*(35) = 3.55, *p* = .001, Cohen’s *d*_z_ = 0.59. Again, however, the cue validity effect associated with the target-color cues was significantly larger than that associated with the irrelevant-color cues, as evidenced by a significant interaction between cue validity and cue color for singleton cue trials, *F*(1, 35) = 4.15, *p* = .049, *η*^2^ = .11. For the feature search task (Fig. 3b), we also found a significant interaction between cue validity and cue color, *F*(1, 35) = 12.28, *p* = .001, *η*^2^ = .26. A significant cue validity effect was associated with the target-color cue, *t*(35) = 5.32, *p* < .001, Cohen’s *d*_z_ = 0.89. In contrast, no cue validity effect was associated with the irrelevant-color cue, *t*(35) = 1.44, *p* = .160, Cohen’s *d*_z_ = 0.24.

### Electrophysiological results

**Fig. 4:**
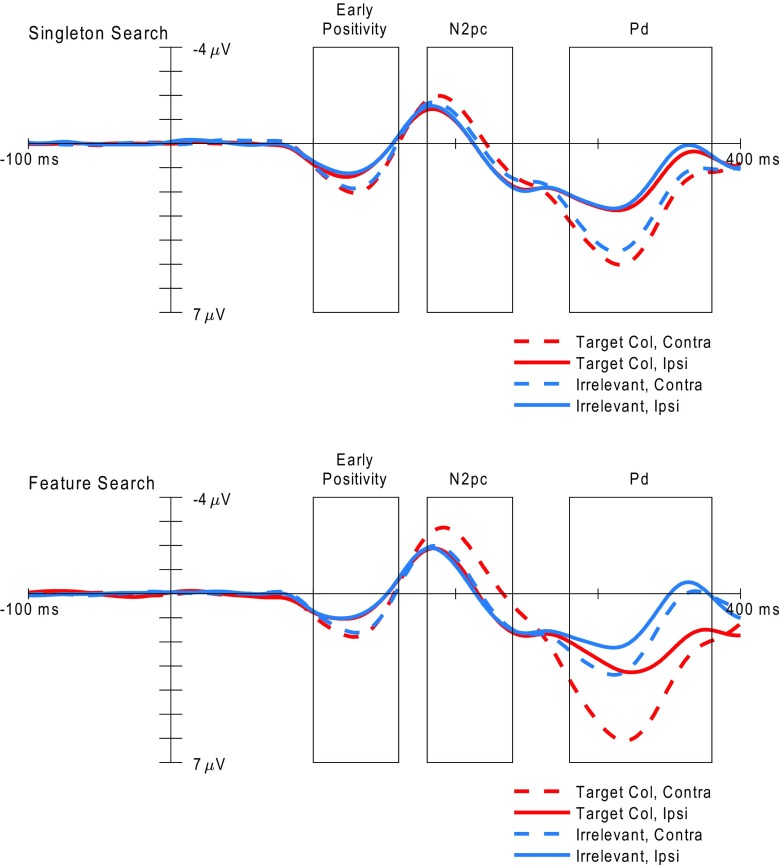
Contralateral and ipsilateral event-related potentials evoked by the cue displays at electrodes PO7/8, separately for each search task and cue color. The three rectangles in each plot indicate the time windows used for the analyses of the early positivity (100–160 ms), N2pc (180–240 ms), and Pd (280–380 ms) components, respectively. Note that negative amplitudes are plotted upward in these plots

**Fig. 5:**
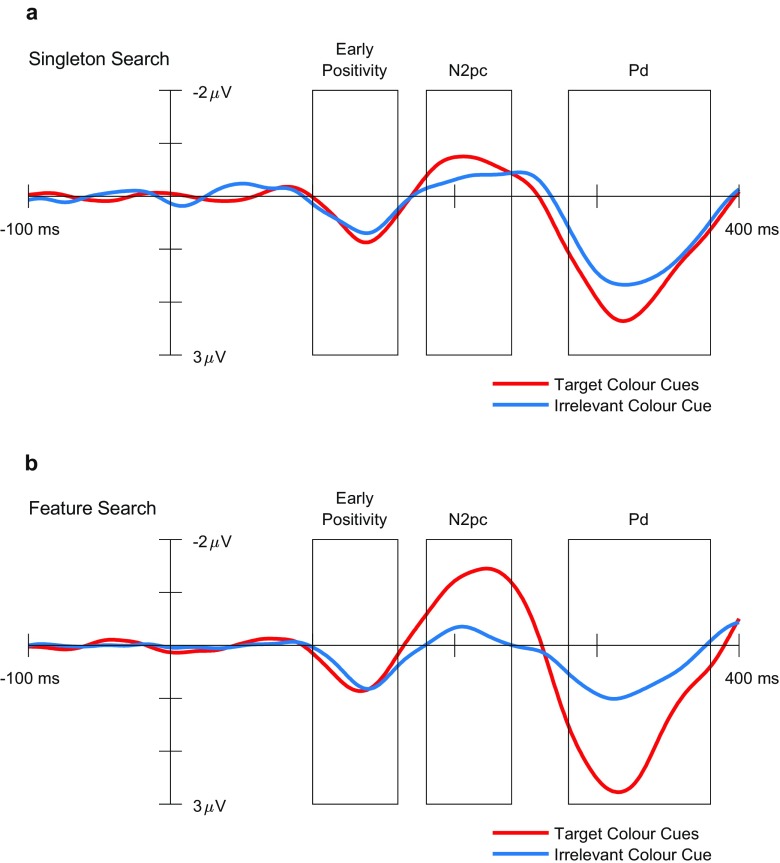
Contralateralized difference waves evoked by the cue displays at electrodes PO7/8, shown separately for each search task and cue color. The three rectangles in each plot indicate the time windows used for the analyses of the early positivity (100–160 ms), N2pc (180–240 ms), and Pd (280–380 ms) components, respectively. Note that negative amplitudes are plotted upward in these plots

The grand average contralateralized ERPs and difference waves observed for each search task and cue color are presented in Figs. 4 and 5, respectively. The contralateralized difference waveforms evoked by the cue displays consisted of an early positive deflection, followed by a negative deflection in the typical time window of the N2pc component (Luck, 2012), and then a late positive deflection. Both the early and late positive deflections fall within the time window during which previous research has reported observing a Pd component (100–400 ms; Sawaki et al., 2012). We chose to define the Pd component as the later of the two positivities observed within this time window, on the basis of research indicating that the Pd tends to occur after the N2pc when both components are present (Sawaki & Luck, 2013). For completeness, we subjected both positivities to statistical analysis. The mean amplitudes for the early positivity (100–160 ms post-stimulus-onset) did not differ as a function of cue color or search condition, producing no significant main effect or interaction, all *F*s < 1. The amplitude during this time window was significantly greater than zero for all combinations of search task and cue color, all *t*s(35) ≥ 5.37, all *p*s < .001, all Cohen’s *d*_z_s > 0.89. These observations are in line with previous evidence that the earliest stages of stimulus processing measurable with EEG tend to be immune to the effects of an observer’s task set (Fellrath, Manuel, & Ptak, 2014).

The mean amplitudes for both the N2pc (180–240 ms) and Pd (280–380 ms) components varied as a function of cue color during both search tasks, as reflected in a significant interaction between search task and cue color: N2pc, *F*(1, 35) = 10.96, *p* = .002, *η*^2^ = .24; Pd, *F*(1, 35) = 12.41, *p* = .001, *η*^2^ = .26. During singleton search (Fig. 5a), target-color cues evoked a significantly larger N2pc component than did irrelevant-color cues, *t*(35) = 2.29, *p* = .028, Cohen’s *d*_z_ = 0.38. The amplitude of the N2pc component was significantly greater than zero for both cue colors [target-color cues: *t*(35) = 5.89, *p* < .001, Cohen’s *d*_z_ = 0.98; irrelevant-color cues: *t*(35) = 2.36, *p* = .024, Cohen’s *d*_z_ = 0.39]. Target-color cues also evoked a significantly larger Pd component than did irrelevant-color cues, *t*(35) = 2.67, *p* = .011, Cohen’s *d*_z_ = 0.45. Again, the amplitude of the Pd component was significantly greater than zero for both cue colors [target-color cues: *t*(35) = 8.45, *p* < .001, Cohen’s *d*_z_ = 1.41; irrelevant-color cues: *t*(35) = 6.90, *p* < .001, Cohen’s *d*_z_ = 1.15].

During feature search (Fig. 5b), target-color cues evoked a significantly larger N2pc component than irrelevant-color cues, *t*(35) = 5.14, *p* < .001, Cohen’s *d*_z_ = 0.86. N2pc amplitudes were significantly greater than zero for target-color cues, *t*(35) = 6.05, *p* < .001, Cohen’s *d*_z_ = 1.01, and irrelevant-color cues, *t*(35) = 2.28, *p* = .029, Cohen’s *d*_z_ = 0.38. There was also a significant effect of cue color on Pd amplitudes, with amplitudes being larger following target-colored cues, *t*(35) = 5.08, *p* < .001, Cohen’s *d*_z_ = 0.85. Pd amplitudes were significantly greater than zero for both cue colors [target-color cues: *t*(35) = 6.92, *p* < .001, Cohen’s *d*_z_ = 1.15; irrelevant-color cues: *t*(35) = 4.03, *p* < .001, Cohen’s *d*_z_ = 0.67].

Comparing N2pc amplitudes between the two search tasks (Fig. 5a vs. b), target-color cues evoked a significantly larger N2pc during feature search than during singleton search, *t*(35) = 4.09, *p* < .001, Cohen’s *d*_z_ = 0.68. This difference suggests that stimuli with task-relevant features capture attention more strongly when the observers’ task set involves a single specific feature value, relative to when their task set includes multiple possible feature values. This possibility is consistent with previous findings that observers can exert attentional control more efficiently when their task set includes a single feature value only, relative to when their task set includes multiple feature values (Barrett & Zobay, 2014; Grubert & Eimer, 2013, 2016; Stroud, Menneer, Cave, & Donnelly, 2012). The magnitudes of the Pd component did not differ between target-colored cues from the feature and singleton search conditions, *t*(35) = 1.08, *p* = .290, Cohen’s *d*_z_ = 0.18.

Surprisingly, comparing N2pc amplitudes between the irrelevant-cue conditions of the two search tasks revealed no difference in the magnitude of singleton capture between the two tasks, *t*(35) = 0.97, *p* = .340, Cohen’s *d*_z_ = 0.16. This result suggests that the emergence of cue validity effects for irrelevant cues under singleton search is not due to enhanced singleton capture. There was, however, a significant difference in the amplitude of the Pd component between the search tasks, with a significantly larger Pd being evoked by the irrelevant cues in the singleton search task than in the feature search task, *t*(35) = 3.37, *p* = .002, Cohen’s *d*_z_ = 0.56, suggesting that the occurrence of a cue validity effect for irrelevant cues under singleton search may be due to increased postcapture inhibition.

## Discussion

In this study, we set out to identify the consequences of singleton detection mode in various stages of stimulus processing. Participants searched for target characters of either any unique color (singleton search task) or one specific color (feature search task). During the singleton search task, a cue validity effect on behavioral responses was evoked by colored cues, regardless of whether the cue color was one of the two possible target colors or a third, irrelevant color. This finding is in line with previous behavioral evidence that observers can set themselves to prioritize any color singleton stimuli (e.g., Eimer & Kiss, 2010; Folk & Anderson, 2010; Harris et al., 2015). Consistent with Eimer and Kiss, we found that the magnitude of the cue validity effect associated with target-color cues was greater than that associated with irrelevant-color cues, suggesting that not all color singletons are given equal priority (Carmel & Lamy, 2015). Importantly, we also demonstrated that the difference between target-color cues and irrelevant-color cues is apparent at the level of electrophysiological measures of both attentional capture (the N2pc component; Luck, 2012) and attentional suppression/disengagement (the Pd component; Sawaki & Luck, 2013).

We also sought to examine whether the presence of a behavioral cueing effect brought about by irrelevant cues in singleton search is evidence of enhanced singleton capture under these conditions or is due to a reduction in the same-location cost (Carmel & Lamy, 2015). Carmel and Lamy (2015) suggested that when a target is a singleton, singleton capture occurs even if the target has a consistent feature. They argued that the effect of singleton capture is often not observed in behavior because it is canceled out by a same-location cost that causes responses to be slower for cued than for uncued targets when the cue and the target have different features. In the singleton search condition, we observed a cue validity effect produced by cues that possessed an irrelevant color, consistent with previous claims from singleton search (Eimer & Kiss, 2010; Folk & Anderson, 2010; Harris et al., 2015). In the feature search condition, however, these same cues produced no cue validity effect, consistent with previous demonstrations from feature search (e.g., Folk & Remington, 1998). If the presence of a cue validity effect produced by irrelevant cues was due to an increase in attentional capture by singleton cues in this condition, we would expect the irrelevant cues in the singleton search condition to also produce a larger N2pc component than those in the feature search condition. In fact, we observed no difference in the N2pc components produced by the two conditions, suggesting that they captured attention to the same extent. This result is inconsistent with the proposal that singleton capture is produced by the instantiation of a singleton set that is not present when participants search for a single feature-singleton target (e.g., a red target among white distractors), or with the presence of a singleton set in search for feature singletons that is enhanced when searching for multiple targets. Instead, the results support an account in which search for multiple features gives rise to cue validity effects for irrelevant features via a reduction in the same-location cost.

The causes of the same-location cost are currently an active topic of investigation. Carmel and Lamy (2014) suggested that the same-location cost may be due to the cost of updating the working memory representation of the item at the target location from possessing the features of the cue to possessing those of the target. Other evidence has suggested that with some stimuli (e.g., spatial frequency), such “object-file” updating does not seem to underlie the same-location cost, whereas with color stimuli it might (Schoeberl, Ditye, & Ansorge, 2018). Our results suggest that the emergence of cue validity effects driven by irrelevant cues in the singleton search condition may be due to a reduction in the magnitude of the same-location cost. Interestingly, this condition was also associated with an increase in the magnitude of the Pd component relative to the irrelevant-cue condition under feature search. Given the strong association between the Pd component and attentional suppression (Hickey, Di Lollo, & McDonald, 2009; Sawaki & Luck, 2010; Weaver, van Zoest, & Hickey, 2017), our results are in line with Carmel and Lamy’s (2015) proposal that the same-location cost is not due to inhibition of capture at the cued location. If this were the case, we would expect to see a larger Pd component in the feature search condition rather than in the singleton search condition. Instead, we speculate that the emergence of a cue validity effect for irrelevant cues in the singleton search condition may have been due to *postattentional* suppression of irrelevant cues in order to prevent their entry into working memory. If the cue is inhibited prior to entering working memory, this may serve to negate the cost involved in updating that working memory representation with subsequent target information.

Another interpretation of the present results is suggested by the recent findings of Livingstone et al. (2017). These authors performed a contingent capture paradigm similar to the one employed here, but with variable target onset times relative to the cue. They observed that the Pd component was locked to target onset, rather than to cue onset, suggesting that rather than being a correlate of attentional suppression, the Pd may actually reflect enhancement of the cued stimulus in the target display. This interpretation is consistent with the present results for the irrelevant cues during singleton search, since both the Pd component and the behavioral capture effect were enhanced under singleton search. However, it is inconsistent with the interpretation implied by the N2pc component. If the Pd component reflects enhancement of target processing due to the capture of spatial attention (reflected in the N2pc), it is unclear why this signal would differ between the feature search and singleton search conditions when the N2pc does not. For this reason we favor the earlier interpretation, though further research will be required in order to empirically test these two possibilities.

One conclusion to be drawn from our findings is that tasks that encourage singleton detection mode do not compel observers to prioritize all singleton stimuli equally (see also Lamy et al., 2006). Instead, it seems that singleton detection mode can be employed in conjunction with other search modes—in this case, a two-feature search mode (Irons, Folk, & Remington, 2012). Under this framework, all singleton stimuli capture attention to some extent, but singleton stimuli with feature values known to be task-relevant (such as the target-color cues in the present study) are given additional priority. This interpretation fits well with models of attention that propose that, rather than being guided by any one aspect of a task or stimulus display, attention is guided by a weighted combination of outputs from a series of priority or activation maps (e.g., Desimone & Duncan, 1995; Wolfe, 1994). The notion that singleton detection mode does not necessarily require giving equal priority to all singleton stimuli is also in line with previous evidence that under certain conditions, observers can prioritize singletons on one feature dimension but not others (Harris et al., 2015; Müller, Reimann, & Krummenacher, 2003).

An important issue raised by the considerations above is whether it is correct to think of “feature search mode” and “singleton search mode” as distinct processes (“modes”), or whether these are simply descriptors applied to different weighting configurations of a unitary attentional guidance system. We believe our results align more closely with the latter possibility. From this perspective, even the formulation of attentional capture as containing separate “target feature” and “singleton capture” components may be misguided in conceptualizing these as separate entities, rather than dynamic weights applied to a multidimensional feature landscape in a way that best differentiates targets from distractors. In such a framework, there would be no distinct search modes for features versus singletons (or for feature relations—Becker, 2010; or for multiple features simultaneously—Irons et al., 2012; or for conjunctions—Becker, Harris, York, & Choi, 2017; etc.), only a weighting of predictive properties that is limited in its effectiveness by the separability of those properties in neural/cognitive feature space. In this framework, the feature maps of such theories of attentional control as Guided Search (Wolfe, 1994) and feature integration theory (Treisman & Gelade, 1980) would not be conceptualized as distinct maps, but rather as axes in a multidimensional feature space in which target weighting can be applied in any number of within- and across-dimensional configurations.

In summary, we investigated the consequences of two task manipulations—a singleton search task and a feature search task—on the processing of identical stimuli. Our findings add to a growing body of evidence (Eimer & Kiss, 2010; Harris et al., 2015) that tasks that encourage singleton detection mode do not necessarily result in all singletons capturing attention equally. Our findings also suggest that the behavioral differences between so-called “feature search” (when the target is a singleton) and “singleton search” may not result from different attentional control settings. Rather, these differences may arise from different postattentive treatments of cue information, resulting in a reduced same-location cost under singleton search. Thus, task performance might be better conceptualized as the result of a dynamic application of multiple search strategies or feature weightings, rather than as the rigid application of a single search “mode.” Future research employing online indices of multiple stimulus weightings will help us understand when, how, and why different combinations of strategies are applied.

### Author note

This work was supported by an Australian Research Council (ARC) Discovery Project to R.W.R. (DP120103721). J.B.M. was supported by an ARC Laureate Fellowship (FL110100103), the ARC Special Research Initiative Science of Learning Research Centre (SR120300015), and the ARC Centre of Excellence for Integrative Brain Function (CE140100007).

## Footnotes

1 Note that other studies have shown that under different conditions, observers are indeed capable of implementing a feature search mode for two distinct feature values (see Beck, Hollingworth, & Luck, 2012; Grubert & Eimer, 2013, 2016; Irons, Folk, & Remington, 2012; Kawahara & Kumada, 2017).

2 The noncolored circles for the first ten participants in the experiment were not gray but white, as had been the case in the study by Folk and Anderson (2010). Thus, for these ten participants only, the cue display was not exactly matched for physical salience across the midline; that is, the colored cue circles had a *lower* luminance than the white circles. Removing these participants from the analyses reported below did not change the patterns of results.
